# Principles and requirements for nanomaterial representations to facilitate machine processing and cooperation with nanoinformatics tools

**DOI:** 10.1186/s13321-022-00669-6

**Published:** 2023-04-12

**Authors:** Kostas Blekos, Kostas Chairetakis, Iseult Lynch, Effie Marcoulaki

**Affiliations:** 1 Institute of Nuclear and Radiological Sciences and Technology, Energy and Safety, National Centre for Scientific Research “Demokritos”, 15341 Agia Paraskevi, Greece; 2 School of Geography, Earth and Environmental Sciences, University of Birmingham, Edgbaston, Birmingham, B15 2TT UK

**Keywords:** Cheminformatics representations, InChI, Nanomaterial identifiers, Nanomaterial structure

## Abstract

Efficient and machine-readable representations are needed to accurately identify, validate and communicate information of chemical structures. Many such representations have been developed (as, for example, the Simplified Molecular-Input Line-Entry System and the IUPAC International Chemical Identifier), each offering advantages specific to various use-cases. Representation of the multi-component structures of nanomaterials (NMs), though, remains out of scope for all the currently available standards, as the nature of NMs sets new challenges on formalizing the encoding of their structure, interactions and environmental parameters. In this work we identify a set of principles that a NM representation should adhere to in order to provide “machine-friendly” encodings of NMs, i.e. encodings that facilitate machine processing and cooperation with nanoinformatics tools. We illustrate our principles by showing how the recently introduced InChI-based NM representation, might be augmented, in principle, to also encode morphology and mixture properties, distributions of properties, and also to capture auxiliary information and allow data reuse.

## Introduction

Nanotechnologies and nanomaterials (NMs) are among the key enabling technologies for European industry, with significant potential for addressing societal challenges in the twenty-first century. During the past decades, there was significant public and private investment to study potential environmental and human health risks of NMs, leading to the development of advanced experimental methods for extensive generation of nanotoxicity data. The rapidly developing field of nanoinformatics covers a variety of computational and decision making tools for data management and the extraction of useful information from available experimental datasets.

In silico modeling includes computational simulations ranging from quantum level, to molecular and coarse grain simulations depicting NM interactions, including agglomerate formation, as well as interactions between NMs and the environment or biological targets. NM characterization, design and discovery have been boosted by machine learning algorithms [1, 2], that take advantage of computational power, high dimension features and big data (when available). Quantitative Structure Activity Relationship models (QSARs) correlate NM properties and behavior according to calculated or measured descriptors of the chemical structure. These models can prove useful for preliminary risk assessment [3, 4] and are increasingly accepted as alternative methods to reduce animal testing. Finally, omics models leverage experimental assays for well-established end-points [5] to study the biomolecular processes involving NMs and link from a Molecular Initiating event through a set of key events to an adverse outcome pathway (AOP) [6].

To support these data-driven models, there has been significant effort during the past decade to create and populate large databases of experimental data produced from industry and research supplemented with calculated descriptors for NMs [7]. Wilkinson et al. [8] provided guidelines for Findable, Accessible, Interoperable and Reusable (FAIR) data, to increase the capacity of machines to access, curate, search, reuse, exchange and analyze large and complex datasets. The European Commission (EC) has invested in coordinated efforts to collect and make available the NM characterization data and biological and toxicological information generated from several large EU-funded projects under the e-NanoMapper database [9]. The e-NanoMapper and the NanoCommons research infrastructure and KnowledgeBase [10, 11] proposed a computational infrastructure for toxicological data management of engineered NMs based on open standards, ontologies and an interoperable design, embracing the FAIR principles and facilitating the development of predictive models [12].

There is a lack of a standardized semantic characterization of NM structural features (such as NM size, shape, number of components and their relations) and environmental variables (such as temperature, medium and other external factors) which are an inseparable part of NM description. This makes it difficult to aggregate, compare, curate and evaluate NM data from different sources and (re)use them for simulations or for training new models.

For conventional chemical substances, web-databases and repositories (e.g., ChEMBL [13], PDBe [14], ZINC15 [15], Pubmed [16] etc.) use standardized linear notations for substance identification (*e.g.*, SMILES [17], SYBYL Line Notation [18], or InChI [19–21]). These notations are also used in deep learning tools [22] to guide the generation of feasible molecular structures featuring desirable properties (e.g., Generative Adversarial networks (GANs) [23], Variational Auto-Encoders (VAEs) [24], Recurrent Neural networks (RNNs) [25] etc.). Extension of e.g., InChI notations have been proposed to consider polymers, mixtures and reactions, and are currently considered for other cheminformatics-relevant components and procedures.

NMs entail additional challenges compared to conventional chemicals. Recently, Lynch et al. [26] initiated an extensive discussion among various stakeholders and proposed a framework for an InChI standard for NMs and a roadmap for its development. They aimed to address the variety of complex nanostructures, using a hierarchical approach which introduces new layers on the InChI notation for the size, shape, crystal structure and ligand binding of the NM, and possibly extrinsic and surface properties.

In the current work we continue and extend this discussion, responding to the need for data normalization, NM description, and protocols for entering, curating and representing data and datasets in NM databases. To achieve this, we present representation assessments on top of which we build concrete extensions that provide standardized methods to represent NMs. Our approach provides the needed framework, according to the principles set out in “Methodology” Section that the representation needs to be accurate, flexible, complete and computable, to enable the systematic building of representations able to depict the complexity of NMs. The proposed principles will allow the construction of representations that should be able to encode all relevant information that a user might need to convey using a higher-level representation. The relevant information, we should note, is not necessarily the most detailed possible, as that is conveyed by, for example, a .mol file (which would include atomic, molecular, crystal structure / unit cell, surface etc.). Such detailed information could not be a substitute for characterization of the NM.

As a proof of concept implementation of our work, in “Results” Section we focus on suggesting layer extensions (e.g., to the morphology and mixture layers) and generic extensions (e.g., for distributions and auxiliary information) to the recently proposed nano-InChI framework developed for accurate encoding and communicating of NMs [26]. We also propose an extension that allows users to create and communicate their own data structures. This enables future-proof solutions for better communication of the intrinsic or extrinsic differences between NM objects, mixtures of NMs/impurities etc. This is important for the development of data-driven approaches for the prediction of NM properties/activities and their correlation.

Finally, by promoting unique representations we aim to facilitate the development of reverse engineering tools for the design of NMs with desirable functionalities, as well as safety and sustainability features under the Safe and Sustainable by design (SSbD) framework [27, 28]. Note that in the case of NMs, the representation should also consider the dynamic nature of real systems, i.e., the extrinsic properties of NMs. This is a very challenging aspect in terms of implementation that has not been considered in past works. “Conclusions and Outlook” Section, discusses how the proposed work can assist the step towards the consideration of non-pristine materials and NM dynamics.

## Methodology

To devise appropriate representation extensions, we first extracted the representation requirements by performing a two-stage analysis in order to (a) define the requirements for a representation that could facilitate the development of data-driven structure-property models, and (b) identify a set of descriptors to focus on and propose extensions to make them more fit for structural representation of NMs and to facilitate machine-based applications.

### Specific objectives and requirements

In this section we identify a set of principles that a NM representation should adhere to in order to provide “machine-friendly” or machine actionable encoding of NMs, that is able to support data management and curation, development and application of modeling tools for phys/chem/bio property prediction, and other machine-oriented operations essential to nanoinformatics. As a whole, an ideal representation should:

- provide a standardized language able to depict the complexity of NMs and able to support similarity assessments and complex search queries in databases;
- be able to encode all relevant NM information at all description levels, meaning that it should accurately describe the core components, shapes, sizes, distribution of those elements, the chemical composition, type of structure or phase and surface characteristics of the NM; and
- be easily extendable by being able to provide—or at least facilitate—future-proof definitions and extensions so that new features can be accurately recorded and communicated, without the need for revising the model.

In this sense, the three crucial features for a machine-friendly NM representation considered here are: accuracy, completeness and flexibility [22, 29–33], as explained below in more detail. In addition, we also consider computability as a desirable characteristic for a machine-friendly representation.

A representation, therefore, following these principles is:

- **Accurate**, when a descriptor can be represented without loss of information, meaning that when we have the representation of a descriptor we can retrieve the descriptor instance exactly as it was before the representation encoding. For example, using a real number to represent average nanoparticle size is an accurate representation. Or, for another example, a .mol or a .xyz file enables an accurate representation of the atoms' position of a chemical substance since all known relevant information can be extracted by the representation without any ambiguities or loss of information. It is evident that the term “accuracy” refers to a specific aspect of the information that is conveyed, so a representation can be accurate in some sense but not accurate in another. In the example of the .mol or .xyz files, these they are not an accurate representation of, for example, the dynamics or the stability of the structure. Since an accurate descriptor does not lose information, this could also cover the need for reversibility. Note that linear chemical identifiers are not always able to fully capture the 3D structure of conventional chemicals [34]. However, NMs comprise a distribution of individual particles (in terms of sizes, surface properties etc.) and, therefore, a distribution of chemical compositions. So, the challenges in nano-identifiers lie beyond the representation of chemical composition and include how to fully capture the 3D structure of the particles or a median representation.
- **Flexible**, when it can encode incomplete information and can be used in different contexts. Therefore, a flexible representation is also incremental, since it should be able to capture information in various stages of completeness without sacrificing the accuracy of the representation. A direct result of this is that a flexible representation can be used as a building block for new representations, for example by reusing small components as parts of bigger new structures or even defining incomplete components as placeholders/generic reusable structures. Therefore, in a sense, flexibility defines successive layers of information-content where the current representation is accurate. For example, the recently proposed NInChI [26] representation is flexible regarding the use of existing InChIs for the small molecule functionalizing ligand. Similarly, but in a much different context, a flexible representation for describing sizes could include layers for increasingly detailed information content such as: knowing the mean size, knowing the general distribution of the sizes, etc.
- **Complete**, if it can encode the entire range of descriptor instances. For example, a representation that encodes nanoparticle shapes using categorical descriptors (e.g., “sphere”, “rod”, “tube”, etc.) is not “complete”, since it cannot capture all possible nanoparticle shapes. Similarly, encoding nanoparticle size using a real number is not “complete” in the sense that a single number is not enough to capture all the nanoparticle size information, which is usually a distribution or a value in some confidence interval, etc.

Two additional important characteristics of a machine-friendly representation are its ability to form general queries, and its computability (or calculability). The combination of those two characteristics enables a wide range of calculations that are of importance to machine operations, and would include similarity calculations to facilitate searches within a database, grouping/read across, pattern extraction etc., as well as quantification of deviations from desired features with respect to shape and size, composition etc. of the NM. To allow the formation of general queries, a representation has to encode the relevant information using a consistent and uniform structure, that is, a structure that stays the same for all—or as many as possible—different realizations of a representation. Moreover, the structure and type of the information encoded should be known before-hand in full detail and the information should be encoded in such a way that retrieval is, ideally, efficient. These calculations would find use, for example, in setting boundaries for nanoforms or sets of nanoforms, or for quality control in materials production e.g., to ensure a NM production is within the allowed deviation from the required characteristics.

Note that we will only consider computability as a required characteristic of a machine-friendly representation. The ability to form general queries is implied, to some extent, by computability as computability enables the ability to form queries of a consistent and uniform structure.

Also, the characteristic of “uniqueness” is not absolutely necessary in order to facilitate machine operations. It is, in general, desirable that representations be unique: two datastructures that represent the same chemical structure should produce identical string outputs. This is achieved by “canonicalizing” the representation; if a chemical structure can be represented more than one way, specific rules are put in place so that only one of these acts as the “valid”/”canonicalized” representation. In the case of NMs, though, the requirement for uniqueness would, among other things, obstruct the incremental properties and increase the complexity of the representation excessively. Therefore, a machine-friendly representation will not guarantee uniqueness and will allow for the possibility that a certain NM corresponds to more than one representation instance. This is not as important as it might seem since (a) it does not apply to the individual components for which already existing, canonical representations are inherited, and (b) the “computability” of machine-friendly representations will allow for similarity comparisons thus making the identification of identical representations and NMs a trivial task.

Finally, a consistent and uniform approach sets the groundwork for creating user friendly representations. Even though user-friendliness is not a requirement for an approach which is good for machine applications, it still needs to be considered as interactions between users and systems are unavoidable and should thus be facilitated. Computability/calculability, on the other hand, is very important for predictive models [35, 36].

### Key challenges to be addressed

The second step of our analysis considers the structural descriptors most commonly used in current NM databases, as well as in simple datasets dedicated to the development of specific prediction models. The e-Nanomapper database [37] is an excellent paradigm for a FAIR and extended database built to support model development. Among the descriptors investigated, we identified those that showed a high usability rate but either failed to accurately describe all use cases or lacked a standardized representation. We then investigated the potential of the InChI implementations, like the recent NInChI [26] on the representation of these descriptors according to the requirements set out in “Specific objectives and requirements” Section. Through this analysis, we identified 11 key challenges, which we grouped into 5 categories according to the strategy used herein to address them.

An important challenge is how to deal with the many missing values or incomplete information in the databases. We consider this in all of the proposed extensions presented in “Results” Section by using incremental representations according to “Specific objectives and requirements” Section.

**Fig. 1:**
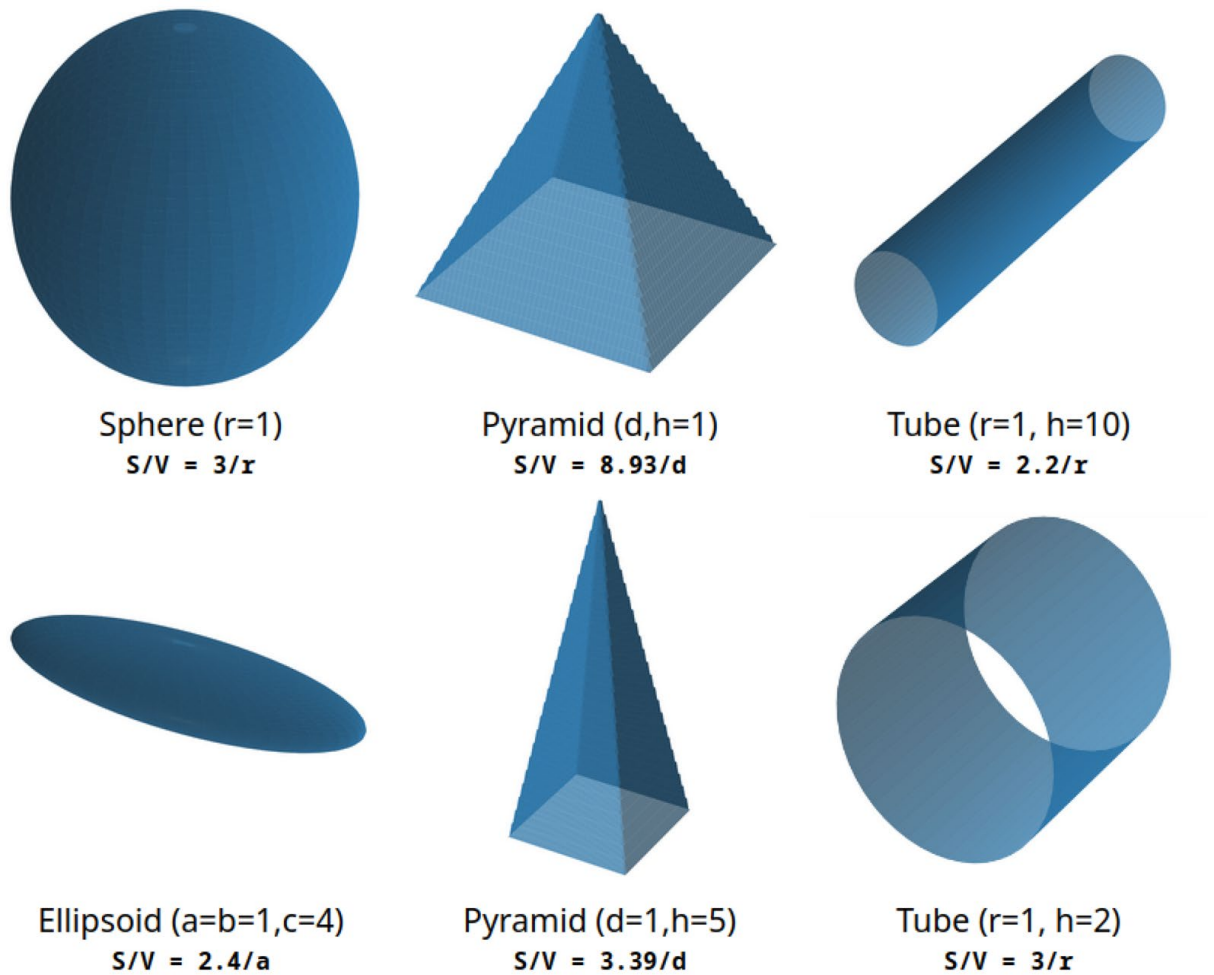
Showcasing the multitude of shapes of NMs and the difficulty of describing and comparing them. Different shapes might share common characteristics, while similar shapes might differ significantly in their parameters. In this figure: sphere/ellipsoid, pyramids, and solid versus hollow tubes

One large category of challenges has to do with the morphology/shape representation of NMs. Shape representation is especially important for NMs but it is also very challenging to construct appropriately. For example, we see it as important to have a generic description of the particle morphology, and not to depend on a set of predefined shapes. Furthermore, the use of categorical variables introduces discontinuities in QSAR models, and makes it difficult to search in databases, quantify structural similarity and investigate morphology-based behavior. NMs of the same shape might not even be directly comparable, or might be more similar to different shapes than to others of the same shape. For example, shapes marked as “rods” might be closer to “nanotubes” or “spheres” than other “rods”, depending on the characteristic one is more interested in (for example surface/volume ratio or main axis ratio, etc.). Figure 1 showcases three pairs of shapes of the same and different morphologies along with metric for their comparison (surface/volume ratio). Other similar issues have to do with the easier extraction of shape similarity metrics enabling user definable morphologies and providing consistent, continuous and comparable ranges/distributions. For all these issues, our general approach, described in “Distributions and ranges” Section and “Morphology” Section, is to use abstract mathematical formulations to describe shape, size and ranges.

The other group of issues that we would like to address have to do with: a) the representation of more complex hierarchies and relations in a way that is consistent with the main principles and requirements we presented, and b) the facilitation of the description of mixtures, defects and, ideally, other impurities present in NMs. We approach these by introducing relevant abstract structures and operators, as described in detail in “Mixtures & Interfaces” Section.

Finally, it is important to include a method that will enable one to capture and query auxiliary information. To address this, we propose the introduction of a structured comment layer as discussed in “Auxiliary info/Comments” Section.

The results of this analysis of issues hampering development of structural representation of NMs are summarized in Table 1, along with the proposed approaches to address these shortcomings. Note that the abstract mathematical formulations to describe shape, size and ranges can consider distributions as discussed in “Distributions and ranges” Section. Regarding the dependence on standard representations, alliance with existing standards is certainly beneficial e.g., for cross-referencing across databases.

**Table 1:** Summary of issues hampering the development of structural representation of NMs in a machine-readable format, and proposed approaches to overcome them

Issue	Proposed approach
Missing or incomplete information	Incremental representations (see “ Specific objectives and requirements” Section)
Description of general shapes	Use abstract mathematical formulation to describe shape, size and ranges
Shapes and Sizes as continuous variables (not categorical)	
Easier extraction of shape similarity metrics	
User definable morphologies	
Consistent, continuous and comparable ranges/distributions	
Represent complex hierarchies and relations	Introduce operators that allow abstract nested structures and flexible hierarchies
Streamline dependency on other InChIs	
Missing description of defects	Introduce modification operators
Capture auxiliary info	Introduce structured comment layer
Query auxiliary info	

In addition to the requirements described in “Specific objectives and requirements” Section, we also consider the following practical issues to extend the applicability potential of our approach. Firstly, we try to keep our results compatible with existing InChI notations, particularly the recent NInChI proposal which aims to become a universal standard for NM notation. Also, the extensions aim to improve the NInChI dependencies on other InChIs, so that we confine the complexity of the representation when mixture or reaction InChIs are used, and promote a more consistent and uniform approach. Note that consistency and uniformity are herein understood in the context of machine implementation: i.e., readable, able to support queries etc. Therefore, a consistent and uniform approach is consistent in the use of keywords, operators and the structure of the representation. Secondly, we explicitly try to focus on extendable approaches, meaning that the proposed definitions should be made as future-proof as possible. This will circumvent the need for specification-setting meetings, improve user-to-user communication, achieve faster communication cycles, and create a NM representation that adapts to specific user needs.

Table 2 provides a summary of the requirements identified as being critical for a NM representation suitable to facilitate machine processing and cooperation with existing and future nanoinformatics tools.

**Table 2:** Summary of requirements for a machine friendly representation of NMs

Primary requirements	Secondary requirements
Accurate	NInChI compatible
Flexible	Reduced complexity
Complete	Extendable/future-proof
Computable/Calculable	

## Results

**Fig. 2:**
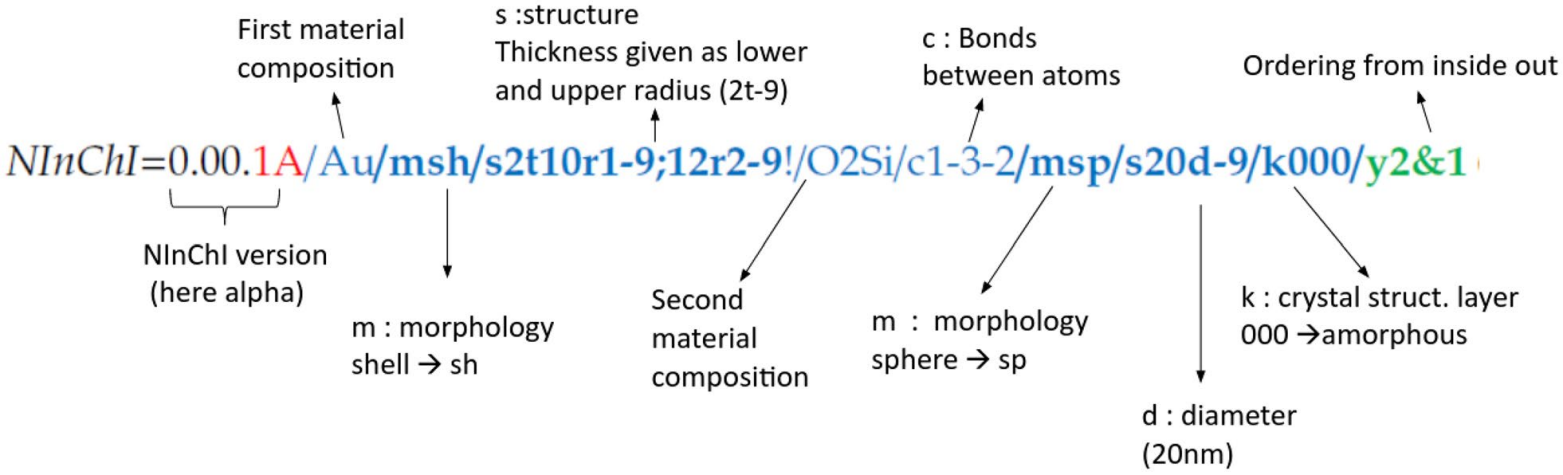
An example of an alpha version (indicated by the 1A) NInChI string that encodes a nanomaterial with two components. Component 1 is a Au shell coating, component 2 is a SiO2 spherical core. Note: the working group on NInChI are aware of some inconsistencies in this approach and its alignment with InChI that will be addressed in the next iteration (beta version or standard extension for nanomaterials 1.0 once accepted by the InChI Trust)

The NInChI proposal [26] extends the InChI representation of chemicals by suggesting new layers aiming to encode specific features of the multi-component structures of nanomaterials. The initial proposal suggests 6 new layers with the first 5 being “sublayers” of the “structural representation layer”. Each (sub) layer includes information related to different properties of a specific component. More specifically, these layers are (Fig. 2):

Composition layer that provides elemental-chemical composition information using the notation inherited from the InChI representation.

- Morphology layer providing information on the morphology of a component.
- Size layer that specifies the size of a component.
- Crystal layer that specifies the crystaline structure of a component, if such a structure can be specified.
- Chirality layer, which, at the moment, is focused on describing the chiral vector (n,m) for carbon canotubes (CNTs).
- Overall structure layer that specifies the overall structure, i.e., the relation between the individual components. This layer is defined as a “top” layer in the initial proposal, unlike the previous sublayers that are part of the “structural representation” layer.

The (sub) layers are separated from each other using the symbol “/”. The components are separated from each other with the symbol “!”. The sublayer is specified by a prefix: ‘m’ for the morphology layer, ‘s’ for the size layer, ‘k’ for the crystal layer, ‘w’ for the chirality layer and ‘y’ for the overall structure layer. Each (sub) layer, then, uses its own “dictionary” to convey the needed information (see Fig. 2 and [26]).

We now present in detail the proposed extensions that aim to address the identified key issues (Table 1) while conforming to the set principles for a structural representation of NMs (Table 2). We propose 5 extensions to the existing NInChI alpha version proposal, as follows:

1. an extension to describe particle distributions; this extension should be available to be applied everywhere where a real number needs to be represented (“Distributions and ranges” Section),
2. an extension to the morphology layer that bypasses categorical variables (“Morphology” Section),
3. an extension of the way that mixtures and interfaces are represented (“Mixtures & Interfaces” Section), based on the introduction of logical operators that apply to other already defined layers. This is built on top of the mixture-InChI [38], which has the goal of facilitating the representation of defects.
4. an extension for structured comments (“Auxiliary info/Comments” Section) that allows consistent and uniform capturing of auxiliary information and facilitates database curation, and finally
5. an extension that allows the definition of macros and shortcuts allowing, thus, the definition of reusable, parametrizable and human-readable components (“Macros & Aliases” Section).

### Distributions and ranges

Most values in NMs are described as ranges or distributions, rather than specific values, for example size distributions, non-uniform charge distributions, non-uniform surface coverage of ligands/polymers etc. Our database investigation highlighted patterns in range and distribution use (Table 3) that we can categorize into two groups. Firstly, we observe large inconsistencies in how distributions are presented, and secondly properties that can be presented as distributions in the database but are not. These properties include all those related to morphology (size and shape) on one hand, and other properties like purity or doping on the other hand. The proposed extension covers both cases in a consistent way. As already discussed in “Methodology” Section, a machine-friendly NM representation should allow for the consistent and uniform description of such ranges through a predefined set of functions that can be easily manipulated. The results of this investigation also illustrated the challenges in searching and integrating data, and the need for harmonization.

**TABLE 3:** Typical distributions and range excerpts found in NM databases

Less than 100 nm
90% is below 20 nm
Size between 60–100 nm
50% < 5 nm, 30% 5–10
Mean 27 nm, stdev 8 nm
15 nm (size distribution 99%)
Mean 115 nm, mode 95 nm

**Fig. 3:**
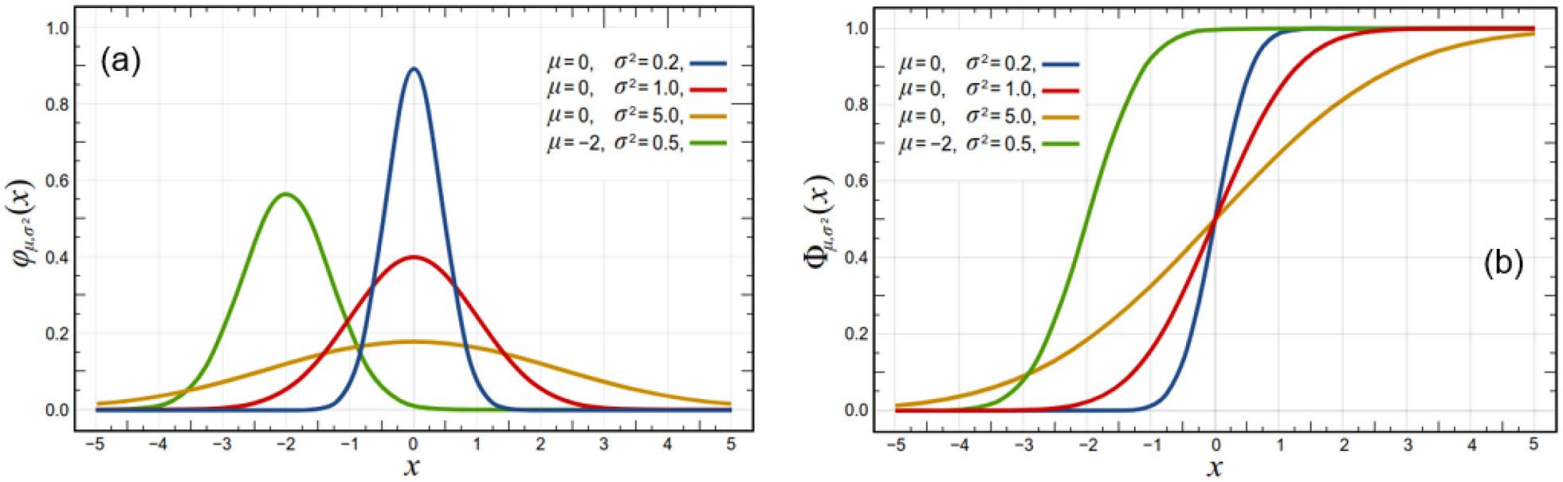
**a** Probability distribution functions (pdf), and **b** their corresponding sigmoid-type cummulative density functions (cdf)

A complete set of such functions should at the very least include a function that can easily describe ranges, such as a sigmoid function (e.g., logistic, error or Heaviside-step function), and a function that can easily describe simple distribution functions from their probability density function (pdf), such as a commonly used pdf (e.g., the normal pdf or the gamma pdf). A sigmoid function (Fig. 3) also allows the description of inequality relationships and can be derived from a cumulative distribution function (cdf) of the respective probability distribution (see Fig. 3).

**Fig. 4:**
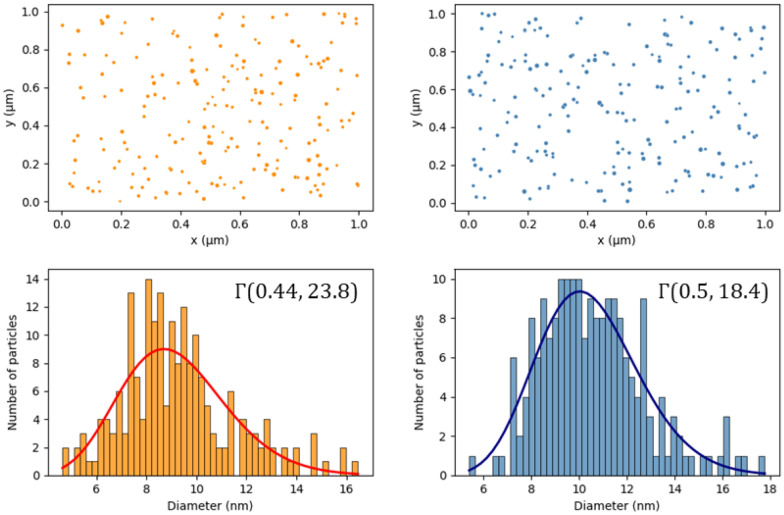
Nanoparticles of various sizes and their respective size distributions. The distributions are fitted to a gamma pdf after calculating the distribution parameters

Such functions have been exhaustively studied and there are many ways to query specific values and ranges, and many ways to compute similarities between them. For example, since the employed distributions are known, one could use metrics such as the Jensen-Shannon divergence or the Kolmogorov-Smirnov distance to measure discrepancy or the similarity between two NMs distributions. Also, it is impossible to make a meaningful comparison of the NM size histogram and TEM images data in Fig. 4 by inspection alone, or just by knowing the mean and standard deviation of the data. By simply including a general pdf fit for the data in the representation, the comparison is straightforward: assuming a gamma distribution fit of Γ(0.44, 23.8) and Γ(0.5, 18.4) respectively, we calculate their Jensen-Shannon divergence at 0.8. General queries then work in a similar manner where a needed feature is calculated as a distance from a desired value, or using geometric means to compare across NMs.

**Fig. 5:**
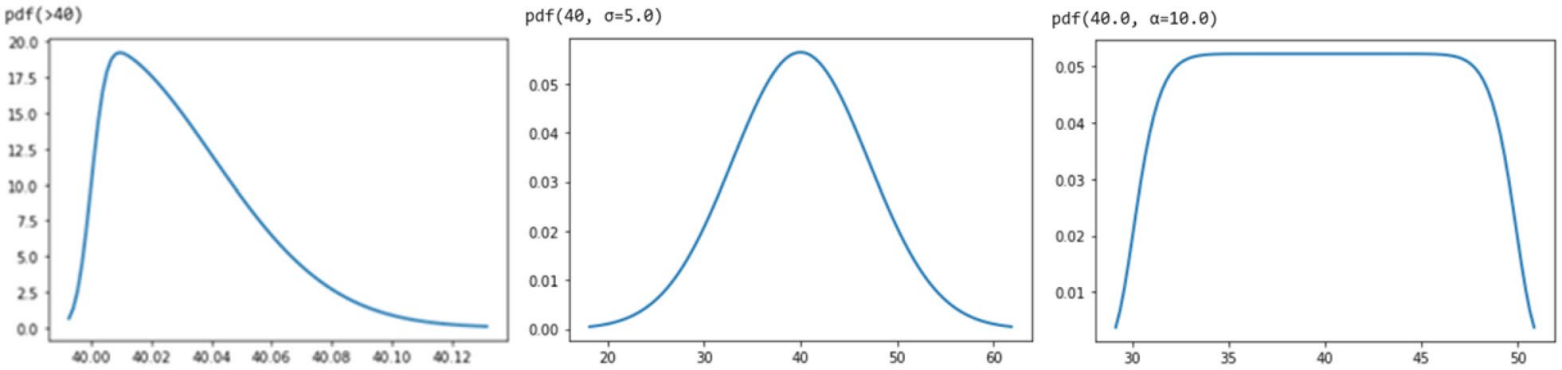
A graphical example of a possible implementation for numbers, distributions & ranges: a pdf for “larger than 40 nm” (left), a specific number that implies a normal distribution (middle) and a range “30–50 nm” (right)

Rules for the combination of the above cdf, pdf etc. functions should also be specified. For example, the functions could be combined via multiplication, addition or other more complex functions. Furthermore, their respective reversed functions and inverse functions could be defined, providing a very flexible framework for describing the necessary distributions and ranges, based on very few components (see Table 4). Table 4 uses a pdf and a cdf function, based on gamma distributions. The known parameters can be specified (for example the confidence interval, the standard deviation or the mean) and the corresponding gamma distribution parameters would be inferred as the best fit to the given parameters. A ‘mixing’ function (denoted by “*” in Table 4) ensures that multiple conditions can be specified simultaneously (e.g. size  ≥ 60 nm and size  ≤ 100 nm). Figure 5 gives a graphical example of a possible implementation.

**Table 4:** Distribution examples using an example set of functions for some pdfs, the respective cdf, the reversed cdf (rcdf) and a mixing function (*). Where parameters are missing, default values are implied that should be defined in the representation implementation

Before	After
Less than 100 nm	$\mathrm{cdf}\left(100\right)$ nm
At least 99.5% purity	$\mathrm{cdf}\left(1, 0.995\right)$→ *use in mixture extension ("Mixtures and Interfaces" Section)*
90% is below 20 nm	$\mathrm{cdf}\left(20, 0.9\right)$ nm
Size between 60-100 nm	$\mathrm{rcdf}\left(60\right)*\mathrm{cdf}\left(100\right)$ nm
50% < 5 nm, 30% 5–10	$\mathrm{cdf}\left(5\right)$ nm, $\mathrm{rcdf}\left(5\right)*\mathrm{cdf}\left(10\right)$ nm *combine with mixture extension ("Mixtures and Interfaces" Section)*
Mean 27 nm, stdev 8 nm	$\mathrm{pdf}\left(27,\sigma =8\right)$ nm
15 nm (size distribution 99%)	$\mathrm{pdf}\left(15,ci=\left(u,0.99\right)\right)$ nm
Mean 115 nm, mode 95 nm	$\mathrm{pdf}\left(115,m=95\right)$ nm

As a proof of concept implementation, it is very easy to extend the layer definitions of the InChI/NInChI framework [26] to include the proposed functions. Below is an example of how the proposed extension can be implemented in the NInChI notation for Au (/Au/) spherical (/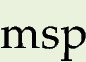/) nanoparticles of diameter 30 nm (/
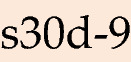
/). Note that the shape description is discussed in “Morphology” Section. The /
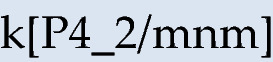
]/ part describes the crystal structure of the core NM by providing the relevant space group information.


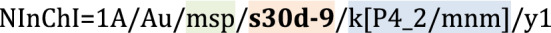
.

We now consider that more information was available but is lost when using the usual representation, such as the diameter of the Au nanoparticles, following a normal pdf with mean 30 nm and standard deviation 2 nm. This can be represented as follows:


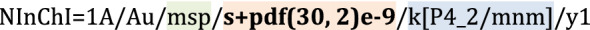
.

Regarding the particle distributions of Fig. 5, they can be represented with the following notations (assuming that they correspond to Au spherical nanoparticles):


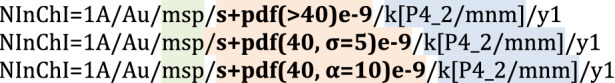
.

### Morphology

During our analysis, it became evident that the shape description was mostly given either qualitatively through visual-inspection or approximately by simple image analysis. At times, a more accurate shape descriptor might be given [39] but in most cases the morphology description was given as a general shape through generic identifiers like “sphere”, “prism”, etc. Table 5 lists some of the most frequently found shapes in NM databases. The requirements of a machine-friendly approach that were set in “Methodology” Section dictate that a morphology representation should use a consistent and uniform way to encode all description cases (however accurate or approximate), ideally in a flexible/incremental method.

**Table 5:** Most frequently found shapes reported in NM databases

Spherical
Irregular
Spherical mixed with irregular
Mixture of spherical, prismatic and rod-shaped
Wire
Triangular or rhombic
Cylindrical
Hexagonal, clubbed
Tubes
Rods
Nanohorn

In the NInChI proposal [26] a NM shape is described by selecting a category from a predefined set of shapes: for example, tube, sphere, shell, etc. In our view this solution is problematic as it is very difficult to predict what will be needed in terms of morphology, and the categorical approach lacks the expressiveness needed to easily and accurately query for shapes and similarities between shapes. It is, therefore, very important that shapes can be abstractly defined. Varsou et al. (2020)[40] proposed geometric mean as a way to compare across particles. TEM images of the particles have also provided the basis to calculate a set of parameters (e.g., boxivity etc.) [41].

In general, it is very difficult to abstractly define shapes in a way that is at the same time accurate, flexible and complete. Additionally, we need a user-friendly representation of the morphology, although this is not strictly necessary to meet the requirements we have set out in “Methodology” Section. However, user-friendliness needs to be considered here since at some point there will be a user that has to manually encode a desired shape, and we deem it important that such interaction should be facilitated.

We propose that all shapes be described by an approximation using a set of predefined functions. Such sets of functions have been studied in depth in the relevant field of digital geometry, where encodings of arbitrary 3D shapes are being studied. NM descriptors could borrow techniques from these digital geometry paradigms or use simplified representations through shape descriptors. Another option is to use a combination of sets of 3D orthogonal functions like the spherical harmonics. In any case, a way to combine functions/methods should be provided to allow for the incremental description of the NM morphology, capturing the appropriate level of detail that is available.

Through the known set of functions, it is now much easier to extract a needed characteristic and compare it between NMs. Calculations of relative volumes or surface/volume ratios, for example, are now straight forward, and other similarity measures can be easily constructed through the functions’ parametrization, to assist general queries.

Table 6 provides examples for the form of possible morphology extensions, using the NM shapes given in Table 5. Here, approximations through expansion to spherical harmonics have been used to describe the shapes. A series of spherical harmonics paired with scaling parameters for each term can be used to approximate arbitrary 3D shapes [42]. In the simplest cases, a series of 1 to 3 terms will suffice. In the InChI/NInChI framework, the proposed functions could replace the categorical values of the morphology layer. Using the example of the spherical Au nanoparticles of the previous subsection, the categorical variable 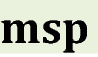 that is set to denote a sphere:

**TABLE 6:** Examples for the form of possible morphology extensions

Before	After
Irregular	Use bounding box distributions
Spherical mixed with irregular	As above + combine with mixtures extension (“Mixtures & Interfaces”Section)
Mixture of spherical, prismatic and rod-shaped	As above + combine with mixtures extension (“Mixtures & Interfaces”Section)
Wire	Y(0,0)*scale(100, 1, 1)
Cylindrical	Y(0,0)*scale(…) + Y(0,1)*scale(…) + Y(0,2) …
Rods	Y(0,0)*scale(4, 1, 1)}
Nanohorn	Y(3, 5)


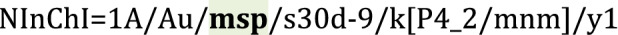
.

can be replaced by using spherical harmonics:


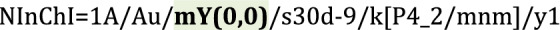
.

Using spherical harmonics enables the calculation of similarity metrics, so we can evaluate the similarity between this nanoparticle and, e.g., an Au rod nanoparticle in terms of their surface to volume or volume ratios characteristics. By combining this approach with the macros & aliases extension, an even more powerful representation becomes possible (see “Macros & Aliases” Section).

### Mixtures and interfaces

The need for descriptions of mixtures of different NMs or the portion of impurities/defects which are ubiquitous, also emerged. Table 7 shows some of the many different ways where a notation for mixing is needed. In the current NInChI proposal [26] the description of mixtures is approached through a dependency on the Mixture-InChI [38] representation while interfaces are not yet implemented. A key difference, though, between NM relation description and mixtures descriptions is that, in the NM case one mostly needs to describe the structure of the relations. Mixture-InChI on the other hand has the goal of capturing definitely what is known about the composition of a given mixed substance. Here we treat mixtures and interfaces as different parts of the same structure since a mixture always has some components and each component always has some relation with the others. Therefore, a successful mixture and interface representation should allow the complete graph representation of the NM structure in an incremental way, describing all known structural information including interfaces, relations-hierarchies and defects.

**Table 7:** Typical descriptions of mixtures, defects and interfaces found in NM databases

(Size) &50% < 5 nm, 30% 5–10
(Size) 90% is below 20 nm
(Shape) mixture of spherical, prismatic and rod-shaped
(Shape) triangular or rhombic
(Media) 0.45% NaCl w/ 0.1% glucose
(Media) mass-concentration proportions (96 mg/L A, 60 mg/L B, 60 mg/L C, 4 mg/L D)
(Defect) at least 99.5% purity

**Fig. 6:**
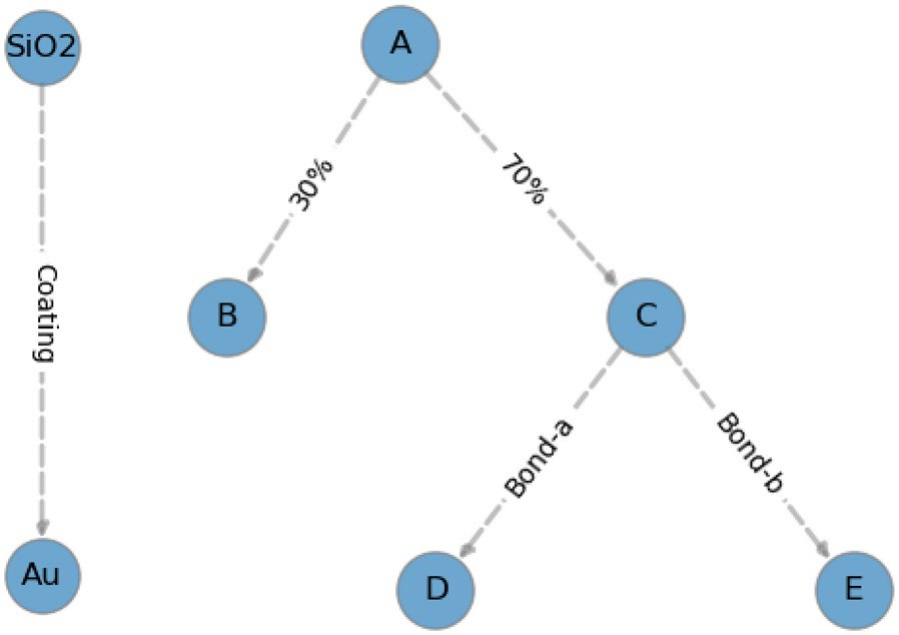
Tree structure using the hierarchy and interface operators. Left: a gold-coated silicon nanoparticle. Right: An abstract nanomaterial with some components “**A**”–”**E**”. Component “**C**” is connected through some “bond-a” to component “**D**”, etc. The percentages 30%, 70% make specific sense in specific contexts; e.g. a mixture with 30% of “**B**” and 70% of the structure “**C**–**D**–**E**”

We propose the introduction of hierarchy and interface operators that can be applied on top of the existing definitions to capture information about component relations, interfaces, bonds, etc. The *hierarchy operators* are representation operators that should at least describe the spatial relation between components so that a “hierarchy” graph can be built as, for example, the structure seen in Fig. 6.

Note that the graph structure on the right of Fig. 6 refers to a Silica coated Au sphere. Using the NInChI framework, it may correspond to NInChI = 1A/Au/msp/s20d-9!/O2Si/msh/s3t-9/y1&2, where the “!” separates the two components, namely component 1 “/Au/msp/s20d-9” and component 2 “/O2Si/msh/s3t-9/”, and the structural layer “/y1&2/” sets the order of the components from the inside-out. The graph structure on the left is an abstract representation of a mixture of two substances or components in a 30:70% mixture; bond information (“Bond-a”, “Bond-b”) is also given for two of the constituents. The *interface operators*, on the other hand, should at least describe the most common possible interfaces—in a way *explaining* the type of hierarchy—between components and other relevant information concerning the relation between components such as bonds, functionalization etc. (represented by the information on the edges of the graph in Fig. 6). Ideally, the definition would be future-proof, allowing the extension of the interfaces that can be described by the user. The defect operators should at least describe the most common possible point defect types, through unions, intersections, etc. The above allows the creation of a graph that describes the structure of the NM. For example, a chemical component and other relevant information could be translated to a graph structure by firstly forming nodes (purple circles in Fig. 6) representing each component. One, then, can define the hierarchy and interface operators as operators that annotate the edges between the nodes, defining the complete structure of Fig. 6. Based on the graph one can easily construct efficient queries as a graph is already a data structure particularly suited for this task.

In terms of implementation, the operators could be realized as incremental descriptors that specify the aforementioned relationships as shown in Table 8. There, the mixture specifications have been replaced by groups of descriptors where the information is now split among the groups. The first group now specifies the main information content (e.g., size, shape or component) while the second group provides the interface information (in this case the mixing percentages) in a one-to-one correspondence. It is already evident that information can be easily processed using this representation; in the first line of Table 8, for example, one can directly confirm that there is missing information for 20% of the input (even if such information can be inferred, it should be noted that it is missing from the original representation).

**Table 8:** Examples of operators used to describe particle distributions in terms of particle size or shape

Before	After
50% < 5 nm, 30% 5–10	{5 nm, 5–10 nm}:{0.5, 0.3}
Mixture of spherical, prismatic and rod-shaped	{sphere, prism, rod}:{}
0.45% NaCl w/ 0.1% glucose	{NaCl, glucose}:{0.0045,0.001}
Mass-concentration proportions:(96 mg/L A, 60 mg/L B, 60 mg/L C, 4 mg/L D)	{A, B, C, D}:{96, 60, 60, 4}mg/L
At least 99.5% purity	…:{$\mathrm{cdf}\left(1,0.995\right)$} → *combine with distributions extension (see "Distributions & Ranges" Section)*

In the InChI/NInChI framework, the layer definitions can be extended to include the proposed operators. The new NInChI layer could be defined to include these operators as a core component or could simply by extended by appending the operators to the structural layer. For example, a two component NM with 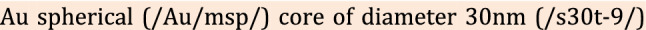 and an 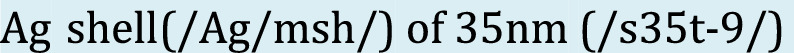, in the NInChI proposal [26] would be written as:


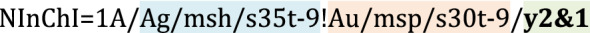
.

The “overall structure” layer 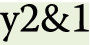 would either be replaced or extended with operators signifying the specific relation of the components. For example, “component 2 includes component 1 with a relation (or bond) of type “b” in a 70:30 ratio”:


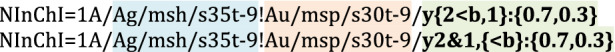
.

### Auxiliary information/comments

NM databases are full of important auxiliary information (Table 9). Such information is usually lost in the representation but it is often crucial and should be both query-able and user-definable so that auxiliary data can be canonically curated, stored and communicated. Table 10 gives some typical examples of defined data types, not confined to NM databases.

**Table 9:** Some of the auxiliary information appearing in the NMs databases

Zeta potential
Test organism
Test conditions
Dates (in various contexts)
Other descriptors

**Table 10:** Examples of defined data types

Data type	Values
Date	ISO date
Float	Real numbers or distributions
Text	Utf-8 string
Units	SI-unit
URI	URLs, ISBNs, DOIs, etc
Enumeration	List of acceptable values

We propose that a method to capture and query auxiliary information should be included in a representation specification. We define a comment structure that includes a formally defined header followed by the content of the comment. The header serves three purposes: (1) it defines the type and structure of the content; (2) it describes the type of the content; and (3) it names this structure so that it can be reused. Ideally, the header could be stored externally so that it is made publicly available for communication and re-use. The existence of a structure alone greatly facilitates queries. It is very easy to search for and convert from the comment structure to an application-appropriate data structure that will make any query easy and efficient. Table 11 provides examples of comment header structures.

**Table 11:** Examples of header structures

Title	Description	Type1[;type2;…]
Zpotential	Zeta potential values; units; URL to medium description	Float;units;uri
Publication	Publication date; Authors; Journal name	Date;text;text
Taxonomy	Subphylum name; Link to taxonomy database	Enumeration;uri

In the InChI/NInChI framework, a comment layer could be defined which would follow our proposed definitions. For example, if we know the zeta potential of a NM:

NInChI = 1A/Au/msp/s30d-9/k[P4_2/mnm]/y1.

it can be included in a comment as follows:


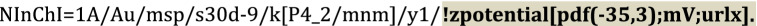


This structured comment makes it very easy to curate, search and find the relevant information. Note that zeta potential is strongly pH and salt-dependent and the solution pH needs to be measured and reported with every zeta potential measurement. This information should be provided at the web address URL that provides the description of the medium, denoted above as “
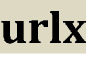
”.

We stress that an InChI implementation of the comment-extension could be made very easily by simply allowing InChI to respect but not define the comment layer structure. The comment structure itself can be specified elsewhere leaving the specification-work to an external entity, instead of amending the InChI specification itself. Table 12 provides example uses of the header structures defined in Table 11, in the comment extension.

**Table 12:** Example uses of the defined header structures that can be included in NInChI specifications

Before	After
Zeta potential	Zpotential[− 35;mV;urlx]
Type of test organism	Taxonomy[crustacean]
Date of publication and authors	Publication[21/10/2019;"Author A., Coauthor B.";"J.Nan"]

### Macros and aliases

A complementary proposition of high importance is the inclusion of a macros & aliases extension that would provide a method to define NMs components that are reusable, parametrizable and human-readable. These can be used to describe complete layers in InChI (e.g., sphere(30e-9)) or specific sub-features of NMs such as crystallographic structures, standardized mixtures, specific doping or defects, or an abstract structure (for example a core-coating structure) etc. This would greatly facilitate future-proof definitions, curation, communication etc. using the NInChI. We propose the inclusion of a macro/alias layer that could contain such extra information as detailed below. In the (N)InChI framework, and in the absence of an appropriate tool-chain that could process such information, this layer should be silently discarded. In an ideal scenario, the appropriate tool-chain will be developed to automatically process and curate this information. The intended workflow would also include pointers to a public database of commonly used alias/macro definitions.

An important goal with the introduction of a macro-layer is to enable one to meaningfully store and/or communicate part of a NM characteristic or feature (NM structure, component, shape, etc.) keeping its structure and making it available for communication and reuse. This way, for example, a partially defined component can be shared as a template or complex shapes can be defined and reused in a database (Table 13).

**Table 13:** Example uses of the macros & aliases extension

Definition	Use
Anatase: = 2O.Ti/k[Ir_1/amd]	Anatase
Sphere(%x): = Y(0,0)*%x	Sphere (30e-9)
Mystructure (%a,%b,%c): = core (%a) coating (%b) sphere (%c)	Mystructure (Au,C8H8,30e-9)

As with the comment layer, in the InChI/NInChI framework, a macro/alias layer could be defined. For example, following our proposed structure to define and replace shape (
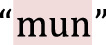
 denotes a morphology yet undefined):


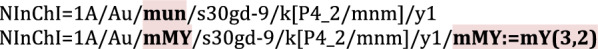
.

or define and replace crystal phase:


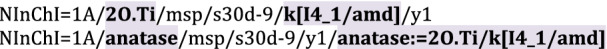


A second driving point for the introduction of a macro/alias layer is opening the door to more general representations encompassing many different descriptors and facilitating NM curation and standardization of representations. A macro/alias extension could serve as a first step towards a programmatic approach to NM representation (see “A language for NM representation” Section).

## Conclusions and outlook

In this work we have analyzed the needs and specified the requirements for future NM representations suitable to facilitate machine processing and cooperation with nanoinformatics tools. At the same time, we have identified in this context, challenges that existing NM representation proposals face, and which the approach described here aims to address. We have laid out the basic principles for an ideal machine-friendly representation. Namely, such a representation should provide a standardized language able to depict the complexity of NMs and support similarity assessments and complex search queries in databases. It should be able to encode all relevant NM information at all description levels and also be easily extendable. We codified these principles into four requirements: a machine-friendly representation should be accurate, flexible, complete and computable.

We also presented specific extensions which can improve existing NM representations on the basis of the principles set here. We have shown how the recently introduced InChI for NMs, in principle, might be augmented through these extensions. The extensions concern the notation layers which define: (a) ranges and distributions, (b) NM morphology, (c) mixtures and interfaces, (d) capturing auxiliary information (comments), and (e) macros and aliases. There are still certain open issues at various levels, for example for the representation of morphologies, and the handling of missing values for mandatory fields, which are key topics currently being discussed in the Nanomaterials InChI Working Group (https://www.inchi-trust.org/nanomaterials/). The incremental approach presented here is the key to addressing these yet enabling implementation immediately.

### Toolchain development

An immediate next goal for this work is to build the appropriate tools that are needed to realize such a representation. The representations discussed in this work are not meant to be handled manually by the users but are designed around an assumed workflow that is based on the existence of appropriate tools. These tools should provide the users with the necessary tool-chain and library support that actually enables the use of such representations. These tools crucially include user interfaces and application programmable interfaces (UI and API) that (a) validate representations, (b) translate between representations and descriptions, and (c) canonicalize representations. Moreover, tools to connect to endpoints, share the representations, etc. would also prove useful to end users and should be considered.

### A groundwork for further discussion

Τhe proposed principles set the groundwork for a useful, universal, NM representation, able to address current challenges in machine processing of NM data. It would be advantageous for a NM representation to be as compatible as possible with other standardized representations like InChI and its extensions to mixtures, reactions and NMs. Thus, the details and specific forms of the proposed extensions need to be discussed with and approved by the NM community and other stakeholders involved in the stages of development, implementation and adoption of a standard notation. To this end, the authors participate actively in relevant discussions as members of the Nanomaterials InChI Working Group (https://www.inchi-trust.org/nanomaterials/).

One important issue to be addressed, specific to NMs and a key component of accurate and correct NM description, is NM dynamics. NMs rarely are only useful or found in their pristine (i.e., as synthesized) forms. NMs constantly evolve through interactions and through their integration with biofluids, the environment etc. Such information is currently available, e.g., in terms of time-resolved data about specific NMs under specific environmental conditions (so-called instances of a NM in the terminology developed by the US CEINT NIKC database which was integrated into the NanoCommons KnowledgeBase [10] to allow aged NMs to be linked to pristine (parent) NMs) [43].

Linear representations usually only consider the pristine forms (as, for example, Lynch et al. [26]) and only represent a frozen-in-time special case of the NM. We are confident that the principles we set out in “Methodology” Section for an accurate, flexible/incremental, complete etc. approach, can provide the theoretical background to gradually capture the real particle dynamics. A lot of decisions will need to be made, so that dynamic aspects are represented efficiently, consistently, accurately, and would enable data processing and extraction of useful knowledge. For instance, consideration is needed on how to include the time dimension within the representation (e.g., using a commonly acceptable discretization of the time domain); how to register additional information on the different media where NMs are dispersed; or how to consider surface stabilizing or functionalizing ligands which can be displaced by proteins or other ligands with higher binding affinities etc. The proposed extensions provide practical solutions for implementation of such cases, e.g., allowing users to: (i) easily define aged NM-variants using parts of their parent structure, and enable the tracking of similarities (common parts) between them; (ii) define mixtures and interfaces for NMs bearing surface-conjugated ligands; (iii) handle variabilities or uncertainties in transformation data e.g., for particle sulfidation, partial dissolution, etc.

### A language for NM representation

An interesting perspective addressing all the above would be the creation of a domain specific language (DSL) for NM representation. A DSL has the “feel” and structure of a general programming language, but is intended for use by specialists in the specific domain (in this case NM experts, handlers, experimentalists etc.) providing tools, methods and language syntax greatly facilitating NM data curation, communication and processing. A DSL would, therefore, incorporate all of the ideas discussed in this work, in a consistent, practical, reusable and extensible programming framework. Based on the requirements set in this work, a DSL would, essentially, form a *lingua franca* for NMs, able to address the peculiarities of NMs description and accessible to experts and non-experts alike. Clearly, the DSL implementation should include interfaces to standard notations.

Following the discussion in “A groundwork for further discussion” Section, the DSL would move further from pristine forms and would enable, at the same time, the consideration of the particle dynamics and hence the time evolution of these systems. For example, by taking advantage of the ideas on hierarchy and interface operators (see “Mixtures & Interfaces” Section), a DSL could offer the tools to describe relationships between different states of a NM and information on the transitions between those states. A DSL, therefore, would extend to the description of NM dynamics, evolution and interactions. Through these, the DSL could greatly facilitate the curation and processing of NM data and, ultimately, play an important role in predicting the properties of and designing new NMs.

## Data Availability

Not applicable.
